# Editorial: Insight in heart valve disease: 2021

**DOI:** 10.3389/fcvm.2022.998862

**Published:** 2022-08-26

**Authors:** Laura Iop, Adrian H. Chester, Roney Orismar Sampaio, Elena Aikawa

**Affiliations:** ^1^Department of Cardiac Thoracic Vascular Sciences and Public Health, Padua Medical School, University of Padua, Padua, Italy; ^2^Magdi Yacoub Institute, London, United Kingdom; ^3^National Heart & Lung Institute, Imperial College London, London, United Kingdom; ^4^Department of Cardiopneumology, São Paulo Medical School, University of São Paulo, São Paulo, Brazil; ^5^Cardiovascular Medicine, Brigham and Women's Hospital, Harvard Medical School, Boston, MA, United States

**Keywords:** heart valve disease, heart valve physiology, heart valve disease management, heart valve disease mechanisms, heart valve bioprostheses

As with many fields of medicine, the last decade of this century has witnessed tremendous progress in the understanding of the physiology, disease mechanism, and the treatment of heart valve disease. The Research Topic entitled “Insights in Heart Valve Disease: 2021” is dedicated to these recent advancements through the original research and perspectives of editorial board members, who seminally contributed to the field.

## Heart valve pathology: Which is its clinical impact in 2021?

Cardiovascular disease is the main cause of mortality worldwide, with several forms of heart valve pathology having a significant contributory role. The article by Hartley et al. proposed an epidemiology study on aortic valve stenosis and demonstrated trends in mortality from aortic stenosis in Europe in 2000–2017 (Hartley et al.). The authors offered an updated overview of the related mortality trends in 23 countries by also considering the impact of transcatheter aortic valve insertion (TAVI) in the last 10 years. Except for Germany and the Netherlands, these trends do not appear declining despite the adopted prevention and care measures. Indeed, they dramatically upraised in some countries of the Eastern European area (e.g., Croatia, Poland, and Slovakia), mostly due to disparities in the clinical practice. Gender still plays a fundamental role: although a greater number of cardiovascular risk factors is generally reported for male patients, females develop more severe aortic stenosis, often difficult to treat even with TAVI due to anatomic-interventional peculiarities. Thus, it emerges a substantial need to revise international guidelines of aortic stenosis care management for an effective, conjoint confrontation of this life-threatening disease.

The article by Perl et al. put the accent on the yet underestimated, long-term impact of ischemic mitral regurgitation through a prospective registry involving more than 3,000 patients in Israel (Perl et al.). This study evidenced the decline of ischemic mitral regurgitation over 20 years but its strong impact on prognosis for diagnosed heart disease patients, who undergo a duplicate one-year mortality risk with respect to the non-diagnosed sub-court.

## Heart valve physiology and pathophysiology: Is our knowledge complete?

Many relevant pieces of the puzzle are still missing in the understanding of the pathophysiology of native valve disease. Original research published in this special issue helps to gain more insights into the mechanism leading to the degeneration of native heart valves. The DNA methylation study performed by Halawa et al. increased the knowledge of both valve biology and disease by the analysis of the left semilunar and atrioventricular valves (Halawa et al.). Almost 600 gene promoters were found differentially methylated in the comparison between aortic and mitral valves and among these, epigenetic modifications were observed in those associated with the WNT-, Cadherin-, Endothelin-, PDGF- and VEGF-pathways, but also the so far less associated TGFB-, NOTCH-, and Integrin-signaling. Consequently, any epigenetic changes occurring in these pathways with relevance for the developmental and pathophysiological events of endothelial-mesenchymal transition (EndMT) and extracellular matrix (ECM) remodeling should also be carefully evaluated during the process leading to the generation of effective, natural heart valve substitutes by tissue engineering. De facto, EndMT reactivation is a potent contributor to valve degeneration even when different pathologies are considered, as shown by Latif et al.. Through TGFB- pathway activation, valve endothelial cells especially acquire a smooth muscle phenotype involving the upregulation of the SM-genes α-SMA, calponin, SM22, SM-myosin, and their co-activators MRTF-A and myocardin in both bicuspid and rheumatic valves, with a phenomenon likely more pronounced in the latter. TGFB- signaling involvement in myxomatous mitral valve degeneration is well renowned, as systematically reviewed in the article by Tang et al.. Mitral valve prolapse manifests in both dogs and humans after myxomatous degeneration with secondary or syndromic forms and is among the major causes of heart failure due to high morbidity and mortality. Although several mechanistic studies have been proposed *in vitro* and *in vivo*, there is still much molecular work to carry out to tackle the disease at its onset by adequate pharmacological targeting instead of surgical interventions.

Another striking original study was advanced by Nasim et al. relating together pigment valve distribution and valve biomechanical functionality (Nasim et al.). By the analysis of aortic valves of transgenic mice with differentially expressed pigments, a proportional increase in elastin content was observed in hyperpigmented animals leading to a surge in stiffness with respect to wild-type animals. On the opposite, hypopigmentation was found to be associated with decreased elastin content and reduced stiffness. Surprisingly, no echocardiographic modifications were found in these different mouse models, as well as no melanocyte cell content could be related to the differential pigment expression. Future experiments will be necessary to investigate more in-depth the possible pathologic link between altered pigmentation levels and valve micromechanical dysfunction.

Biomechanical impairment is widely recognized as a causal event in atrioventricular valve degeneration, too, although only secondary again to an upregulation of TGFB- signaling. Vinciguerra et al. focused on this molecular valve dysfunction culprit in their two review articles (Vinciguerra et al.; Vinciguerra et al.). The mechanical stress generated by mitral plasticity can be counteracted by the surgical approach of subvalvular correction reassuring an appropriate leaflet coaptation. Pharmacological treatments, such as renin-angiotensin-aldosterone system (RAAS) inhibitors, demonstrated to be equally effective, by contrasting valve remodeling consequent to acute myocardial infarction. Among these drugs, losartan showed to be promising, also proving a strong ability in blocking EndMT in valve endothelial cells and valve fibrosis. A minor medical interest has been relieved over time by tricuspid regurgitation, which, however, is increasingly documented as strongly relevant for its impact on cardiovascular disease prognosis. This novel awareness has rendered possible a greater characterization of the tricuspid regurgitation subtypes, due to right ventricle overload and tricuspid annular dilatation resulting from left heart valve disease. The application of three-dimensional echocardiography imaging through a transthoracic window was demonstrated to be efficacious in the diagnostic course and severity stratification, while cardiac magnetic resonance rendered finally feasible quantitative studies on right heart remodeling, and particularly associated valve regurgitation.

## Heart valve disease: Are current therapies successful in the long-term?

Surgical and mini-invasive treatments are becoming more targeted and effective, also with reference to the management of electrical conduction abnormalities. Valve correction, as well as substitution with bioprosthetic replacements, have reached a routinary application for the therapeutic management of heart valve disease. In their 1-year PERSIST-AVR clinical trial, Fischlein et al. evaluated the performance of one of the most recent bioprosthetic valve concepts, i.e., sutureless replacements, with respect to conventional ones in the treatment of aortic valve regurgitation (Fischlein et al.). This study disclosed a non-inferiority of the Perceval sutureless valve compared to stented sutured counterparts in several hemodynamic parameters, such as pressure gradients, valve effective orifice area, and paravalvular leak.

Nevertheless, there are still some unsolved flaw in the usage of animal-derived valve bioprostheses and their dystrophic evolution in the mid/long term. In this regard, several hypotheses were proposed over time, as emphasized by Human et al. in their review article (Human et al.). In the last years, several pieces of evidence are corroborating the notion of immunogenicity-related valve degeneration. Although different technologies are finding applications to possibly reduce this animal burden (from simple alcohol-mediated glycolipid washout, passing through decellularization, to more sophisticated animal genetic engineering to silence or modify the signaling relevant for the expression of sugar xenoantigens), the studies performed in animal models likely suggest that something is still beyond the comprehension of the link between various xenoantigens and a sustained immune reply by the host. Fascinatingly, this review motions a further standpoint: xenoantigenicity might be not only related to the peculiar sugar moiety intrinsic to animal cells, their remnants, and/or extracellular matrix but also tissue bioburden. Sterilization protocols, standardly employed for medical devices, might be too detrimental for natural tissues to be employed for replacement manufacturing, thus maintenance of microorganisms, such as bacteria and viruses, might be probable after the application of milder decontamination treatments. This residual bioburden might match a remaining load of the same xenoantigens endowed in the animal tissues since several colonizing bacteria expose these glucidic groups on their cell membranes. In the support of this hypothesis, a parallel between bioprosthetic dystrophic calcification and rheumatic fever is provided in the review to point out the potential association of inflammation, pannus overgrowth, and/or autoimmunity observed in the progress of both diseases. While more studies are compulsory to confirm this, a quest for more effective sterilization treatments for natural tissues is undeniable.

In their original article (Pietrasanta et al.). Apart from the substitutes intended for classic surgery, the last decades have been characterized by the incredible technological revolution in valve disease therapy led by mini-invasive transcatheter replacement approaches, more commonly TAVI when intended for aortic valve treatment. The undisputable benefit for high mortality risk patients in advanced age might lessen its strength in younger subjects, for which a longer-term evaluation is foreseen. Possible dysfunctions might be associated again with the proneness to inflammatory/immune degeneration in those TAVI devices produced with animal tissues. Pietrasanta et al. reflected on the detrimental effects caused by the turbulent flow induced by different device deployment modalities. This wet-lab research performed through the use of a pulse replicator allowed to typify the three-dimensional flow field generated by different implantation configurations of a self-expandable TAVI valve in porcine pericardium. Such *in vitro* experimental analyses are essential to simulate the pathological peculiarities of the patient's root and, thus, their results are strongly informational for the best deployment strategy to use and for the prevision of the functional behavioral fate of TAVI devices, once implanted in the patient.

In addition, cardiac hemodynamic load and particularly valve regurgitation might find relief in most patients when their comorbidities are clinically treated, as proved by Kim et al. in their original research article focusing on left heart valve regurgitation and the favorable effects exerted by renal functional improvement after transplantation (Kim et al.). Interestingly, in a small percentage of patients (<10%), mitral and aortic regurgitations might progress although the initial success of the kidney transplantation and lead to the need for a second procedure due to the maintenance of a hypertensive state, valve calcifications, and reduced left ventricle (LV) end-systolic dimensions.

## Take-home message

As a conclusive remark, all the original and reviewed knowledge reported in this Research Topic has shed more light on the physiology and pathophysiology of heart valve disease and associated cardiac and non-cardiac pathologies (Table 1). Based on these advances, it is evident that the standard of clinical care, as well as novel therapeutic approaches, should be fine-tuned thereof worldwide to have a more positive impact on the natural history and prognosis of cardiovascular diseases, the life quality of affected patients, and socio-economical health management programs.

**Table 1:** Take-home messages from the Research Topic ‘Insight in heart valve disease: 2021'.

**Heart valve disease management**	**Article title**	**Key points**	**Possible implications**	**Reference**
Heart valve disease epidemiology	Trends in Mortality From Aortic Stenosis in Europe: 2000–2017	• Increasing mortality rates from aortic stenosis in Europe • Women develop more severe aortic stenosis than men • European countries have different standards of care	• Need to substantially revise international guidelines of aortic stenosis care management	Hartley et al.
	Trends in Ischemic Mitral Regurgitation Following ST-Elevation Myocardial Infarction Over a 20-Year Period.	• Ischemic mitral regurgitation has declined over 20 years. • However, diagnosed patients have a double mortality risk than non-diagnosed ones.	• Strong impact on prognosis	Perl et al.
Heart valve (patho)physiology	Profiling Genome-Wide DNA Methylation Patterns in Human Aortic and Mitral Valves.	• 600 gene promoters were found differentially methylated between aortic and mitral valves • Epigenetic modifications were observed in those associated with the WNT-, Cadherin-, Endothelin-, PDGF-, VEGF-, TGFB, NOTCH, and Integrin signaling.	• Epigenetic changes in these pathways, relevant for EndMT and ECM remodeling, should be carefully evaluated during heart valve tissue engineering.	Halawa et al.
	Atypical Expression of Smooth Muscle Markers and Co-activators and Their Regulation in Rheumatic Aortic and Calcified Bicuspid Valves.	• Upon TGFB- pathway activation, valve endothelial cells acquire a smooth muscle phenotype with upregulation of the SM-genes and their co-activators in both bicuspid and rheumatic valves	• EndMT reactivation contributes to valve degeneration in both bicuspid and rheumatic valves	Latif et al.
	The Role of Transforming Growth Factor-β Signaling in Myxomatous Mitral Valve Degeneration.	• Mitral valve prolapse manifests in dogs and humans after myxomatous degeneration. • It is among the major causes of heart failure due to high morbidity and mortality. • Several mechanistic studies *in vitro* and *in vivo* have been proposed.	• Pharmacological targeting is still missing.	Tang et al.
	Pigmentation Affects Elastic Fiber Patterning and Biomechanical Behavior of the Murine Aortic Valve	• Pigment content increases proportionally with elastin in heart valves • No echocardiographic modifications were appreciated. • No melanocyte cell content was correlated to pigmentation levels.	• Further studies are mandatory to understand the effect of pigmentation on valve micromechanical (dys)function.	Nasim et al.
	Mitral Plasticity: The Way to Prevent the Burden of Ischemic Mitral Regurgitation?	• Mechanical stress generated by mitral plasticity can be counteracted by surgical subvalvular correction • Pharmacological treatments, such as RAAS inhibitors (e.g., losartan), contrast valve remodeling consequent to acute myocardial infarction.	• Blocking EndMT in valve endothelial cells and valve fibrosis is effective to prevent mitral plasticity.	Vinciguerra et al.
	Functional Tricuspid Regurgitation: Behind the Scenes of a Long-Time Neglected Disease.	• Tricuspid regurgitation has a strong impact on cardiovascular disease prognosis.	• Three-dimensional echocardiography allows for severity stratification.	Vinciguerra et al.
		• Characterization of the tricuspid regurgitation subtypes is now possible by current sophisticated imaging. • Cardiac magnetic resonance renders feasible quantitative studies on right heart remodeling, and associated valve regurgitation.	
Heart valve disease treatment	Hemodynamic Performance of Sutureless vs. Conventional Bioprostheses for Aortic Valve Replacement: The 1-Year Core-Lab Results of the Randomized PERSIST-AVR Trial.	• Perceval sutureless valves show good pressure gradients, valve effective orifice area, and paravalvular leak.	• A non-inferiority of the Perceval sutureless valve compared to stented sutured counterparts is disclosed in terms of haemodynamic performances.	Fischlein et al.
	Residual Bioprosthetic Valve Immunogenicity: Forgotten, Not Lost.	• Valve degeneration might be immunogenicity-related. • Residual bioburden might induce immunogenic responses by the host toward the xenoantigens displayed by colonizing bacteria. • Bioprosthetic dystrophic calcification and rheumatic fever might parallel in their manifestations.	• Further analyses are needed to establish whether residual bioburden might be causal to bioprosthetic valve degeneration. • If this hypothesis is valid, improvements in terminal sterilization protocols are compulsory.	Human et al.
	Characterization of Turbulent Flow Behind a Transcatheter Aortic Valve in Different Implantation Positions.	• Turbulent flow induced by different TAVI device deployment modalities can be studied in pulse replicator to typify the three-dimensional flow field generated.	• Such *in vitro* experimental analyses are essential to simulate the pathological peculiarities of the patient's root. • Their results are informational for the best deployment strategy to use and for the prevision of the functional behavioral fate of TAVI devices.	Pietrasanta et al.
	Mitral and Aortic Regurgitation in Patients Undergoing Kidney Transplantation: The Natural Course and Factors Associated With Progression.	• Renal functional improvement after transplantation generally exerts favorable effects on left heart valve regurgitation. • In less than 10% of patients, mitral and aortic regurgitations might progress.	• It is necessary to investigate why a few patients with initial successful kidney transplantation require a second procedure due to the maintenance of a hypertensive state, valve calcifications, and reduced LV end-systolic dimensions.	Kim et al.

End-MT, endothelial-mesenchymal transition; ECM, extracellular matrix; SM, smooth muscle; RAAS, renin-angiotensin-aldosterone system; TAVI, transcatheter aortic valve insertion; LV, left ventricle.

## Author contributions

LI conceived and wrote the editorial. AC, RS, and EA wrote and approved the editorial. All authors contributed to the article and approved the submitted version.

## Conflict of interest

The authors declare that the research was conducted in the absence of any commercial or financial relationships that could be construed as a potential conflict of interest.

## Publisher's note

All claims expressed in this article are solely those of the authors and do not necessarily represent those of their affiliated organizations, or those of the publisher, the editors and the reviewers. Any product that may be evaluated in this article, or claim that may be made by its manufacturer, is not guaranteed or endorsed by the publisher.

